# Fused-Ring Derivatives of Quinoxalines: Spectroscopic Characterization and Photoinduced Processes Investigated by EPR Spin Trapping Technique

**DOI:** 10.3390/molecules190812078

**Published:** 2014-08-12

**Authors:** Zuzana Barbieriková, Dana Dvoranová, Maroš Bella, Viktor Milata, Adriana Czímerová, Vlasta Brezová

**Affiliations:** 1 Institute of Physical Chemistry and Chemical Physics, Faculty of Chemical and Food Technology, Slovak University of Technology in Bratislava, Radlinského 9, Bratislava SK-812 37, Slovak Republic; 2 Institute of Chemistry, Slovak Academy of Sciences, Dúbravská cesta 9, Bratislava SK-845 38, Slovak Republic; 3 Institute of Organic Chemistry, Catalysis and Petrochemistry, Faculty of Chemical and Food Technology, Slovak University of Technology in Bratislava, Radlinského 9, SK-812 37 Bratislava, Slovak Republic; 4 Institute of Inorganic Chemistry, Slovak Academy of Sciences, Dúbravská cesta 9, Bratislava SK-845 36, Slovak Republic

**Keywords:** quinoxalines, photochemistry, EPR spectroscopy, spin trapping, UV/vis, fluorescence spectroscopy

## Abstract

10-Ethyl-7-oxo-7,10-dihydropyrido[2,3-*f*]quinoxaline derivatives, synthesized as promising biologically/photobiologically active compounds were characterized by UV/vis, FT-IR and fluorescent spectroscopy. Photoinduced processes of these derivatives were studied by EPR spectroscopy, monitoring *in situ* the generation of reactive intermediates upon UVA (λ_max_ = 365 nm) irradiation. The formation of reactive oxygen species and further oxygen- and carbon-centered radical intermediates was detected and possible reaction routes were suggested. To quantify the investigated processes, the quantum yields of the superoxide radical anion spin-adduct and 4-oxo-2,2,6,6-tetramethylpiperidine *N*-oxyl generation were determined, reflecting the activation of molecular oxygen by the excited state of the quinoxaline derivative.

## 1. Introduction

Nitrogen-containing heterocyclic compounds have recently gained the intense attention of a wide scientific audience due to their diverse applicability and easy synthesis routes [1,2,3,4]. Substituted quinoxalines have always been linked with a broad spectrum of biological activities. These benzoheterocycles constitute a wide range of pharmacological active compounds possessing antibacterial, antifungal, anticancer, antitubercular, antimalarial and antidepressant activities [5,6,7,8]. Moreover the planar conformation of the quinoxaline heterocycles enables their interactive binding with DNA [1,9]. Consequently the research in this field is mainly focused on the structural modification of the quinoxaline derivatives in order to control their therapeutic properties. In addition, many pharmaceutically utilized heterocyclic compounds are known to potentially lose or increase their biological activity upon UV exposure [10,11,12,13,14]. These processes can be related to the generation of reactive oxygen species (ROS) via the molecular oxygen activation by the excited states of the photoactive molecule or directly by the molecular changes in the compound itself [13,15,16,17,18]. Therefore the knowledge on the UV/vis light sensitivity of these compounds is important for their further applications in biological systems. Beside these indisputable biological/photobiological effects, compounds containing the quinoxaline moiety find their applications as fluorescent sensors, stains for microscopy and diagnosis in medicine or in optoelectronic devices [1,2,3,19,20]. Incorporation of an *N*-heterocycle such as quinoxaline, characteristic with the highly π-electron deficient nitrogen atoms, in the backbone of luminescent molecules significantly modifies the photophysical properties of π-conjugated materials. Moreover, the presence of nitrogen atoms with lone electron pairs allows the quinoxaline ring to act as an effective and stable complexing agent [2].

**Figure 1:**
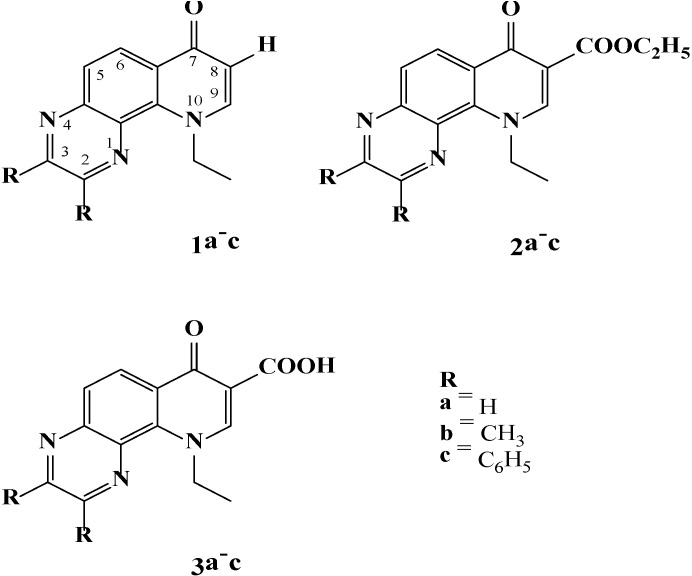
10-Ethyl-7-oxo-7,10-dihydropyrido[2,3-*f*]quinoxalines investigated.

The large application potential of heterocyclic compounds containing the quinoxaline moiety motivated the preparation of new quinoxaline derivatives [8,21,22,23,24] and their further characterization. We focus attention on the photoinduced processes of 10-ethyl-7-oxo-7,10-dihydropyrido[2,3-*f*]-quinoxalines (Figure 1) within the scope of their photoactivity, as the first step in the characterization and selection of the promising candidates for photobiological or luminescent applications. EPR photochemical experiments enable us to monitor the generation of reactive intermediates *in situ* by various well established and widely applied indirect methods. The EPR spin trapping technique provides the identification and quantification of the radical species generated in the studied systems upon photoexcitation. Since UV/vis irradiation of heterocyclic compounds under air often leads to molecular oxygen activation coupled with the generation of reactive oxygen species, singlet oxygen formation was also monitored indirectly by the oxidation of sterically hindered amines to the semi-stable nitroxide radicals.

## 2. Results and Discussion

### 2.1. UV/vis and Fluorescence Spectroscopy

**Figure 2:**
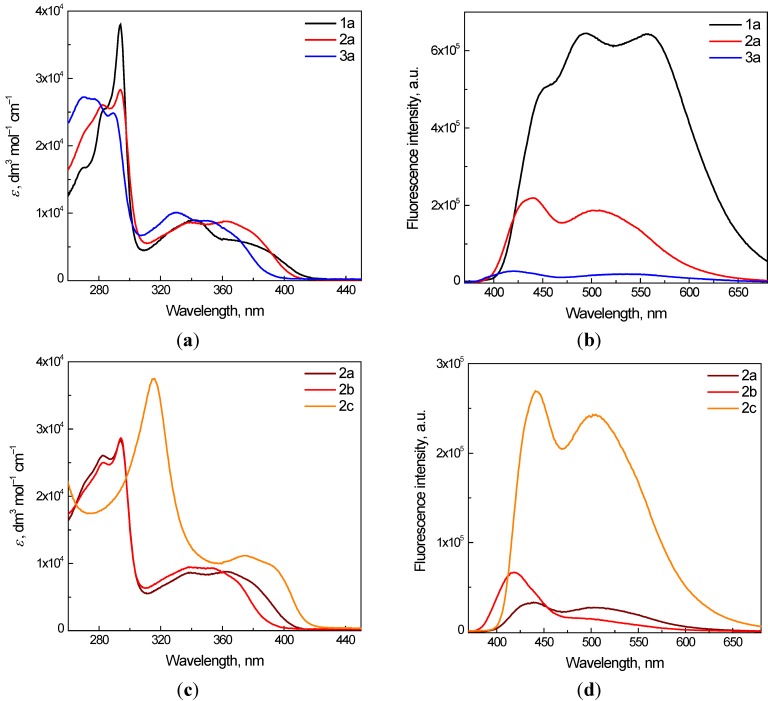
(**a**,**c**) Electronic absorption spectra of selected quinoxaline derivatives **1a**–**3a**, **2a**–**c** in dimethylsulfoxide (DMSO); (**b**,**d**) Fluorescence emission spectra of selected quinoxaline derivatives **1a**–**3a**, **2a**–**c** in DMSO under air. Excitation was performed at 350 nm.

The electronic absorption spectra of the synthesized quinoxalines (Figure 1) were measured in the aprotic solvent dimethylsufoxide (DMSO) or in the mixed solvent DMSO/water (1:1 v:v), due to the limited solubility of the derivatives in water. The absorption maxima obtained, along with log of molar absorptivities are summarized in Table 1. UV/vis spectra of the 10-ethyl-7-oxo-7,10-dihydropyrido[2,3-*f*]quinoxalines **1a**, **2a** and **3a** with varying substitution at the C-8 (Figure 2a) show only a small effect of the substitution character on the position of the absorption maxima. The unsubstituted 10-ethyl-7-oxo-7,10-dihydropyrido[2,3-*f*]quinoxaline **1a** reveals three absorption maxima at 294, 342 and 380 nm, which can be attributed to the electron transfer from the 4-quinolone to quinoxaline moiety as was supported by theoretical calculations, which also evidenced only a negligible effect of π-electrons from the phenyl groups in the non-planar position for derivative **1c** (Figure S1). In the absorption spectra of substituted **2a** and **3a** a decrease in the intensity of the high energy absorption band at 294 nm and a minor effect on the location of the low-energy absorption bands is evident. A slight increase of the maxima at 376 and 361 nm can be observed for the ethyl carboxylate **2a** while the absorption peaks of the carboxylic acid **3a** are shifted to 353 and 331 nm. A more intense effect on the electronic absorption spectra appears for the di-substitution at C-2 and C-3 (Figure 2c). While the absorption spectra of **2a** and **2b** show only small differences in the position of the absorption bands in the region 300–400 nm, for 2,3-diphenyl derivative **2c** intensive absorption peaks with maxima at 316 nm, 375 and 395 nm were found. Due to the poor solubility of the studied derivatives in water, mixed solvent DMSO/water (1:1 v:v) was used, and in the case of derivative **3c** addition of NaOH was necessary for the solubilization. The effect of the solvent exchange on the electronic absorption spectra of the quinoxaline derivatives demonstrate the values of the absorption maxima positions and corresponding log of molar absorptivities listed in the Table 1. A slight hypsochromic shift of the low-energy absorption bands can be observed in the mixed solvent, reflecting the exchange of the aprotic solvent DMSO with DMSO/water (1:1 v:v).

**Table 1:** UV/vis absorption maxima with log of molar absorptivities (in italics) of the quinoxalines investigated in dimethylsulfoxide and dimethylsulfoxide/water (1:1 v:v) mixed solvent.

Compd.	λ_max_ (nm)/log *ε*λ_,max_(dm^3^·mol^−1^·cm^−1^)	
	DMSO	DMSO/Water (1:1 v:v)
**1a**	380 ^sh^/3.726, 342/3.955, 294/4.580	373 ^sh^/3.540, 334/3.898, 290/4.414, 268/4.200
**1b**	370 ^sh^/3.663, 342/3.905, 295/4.541, 284/4.327, 270/4.195	367 ^sh^/3.603, 332/3.960, 292/4.488, 281/4.349, 270/4.314
**1c**	385 ^sh^/3.887, 318/4.616	390 ^sh^/3.778, 375/3.885, 317/4.531
**2a**	376 ^sh^/3.880, 361/3.945, 340/3.938, 294/4.452, 283/4.415	376 ^sh^/3.742, 356/3.878, 333/3.915, 291/4.369, 279/4.365
**2b**	370 ^sh^/3.854, 354/3.965, 338/3.977, 294/4.457, 283/4.397	367 ^sh^/3.773, 351/3.894, 332/3.923, 291/4.377, 279/4.347
**2c**	395 ^sh^/3.973, 375/4.048, 316/4.574	390 ^sh^/3.970, 372/4.030, 314/4.510
**3a**	368 ^sh^/3.826, 353/3.943, 331/4.005, 290/4.393, 279/4.431, 270/4.435	368 ^sh^/3.812, 351/3.949, 331/4.005, 291/4.391, 279/4.429, 270/4.435
**3b**	372 ^sh^/3.859, 352/4.012, 331/4.070, 290/4.458, 278/4.493, 271/4.496	367 ^sh^/3.905, 351/4.015, 330/4.070, 290/4.456, 278/4.491, 271/4.496
**3c**	^#^ 420 ^sh^/3.437, 390/3.943, 370/3.960, 315/4.341	^&^ 400 ^sh^/3.677, 386/3.872, 375/3.892, 316/4.388

^sh^: shoulder; ^#^: contains equimolar amount of NaOH in DMSO/water (200:1 v:v), ^&^: contains an equimolar amount of NaOH.

The quinoxaline moiety is considered as representative fluorophore and quinoxaline derivatives are often applied as sensitive fluorescent probes [1,2,25,26]. The fluorescence emission spectra of 10-ethyl-7-oxo-7,10-dihydropyrido[2,3-*f*]quinoxaline derivatives measured in aerated DMSO solutions at the excitation of 350 nm are influenced by the character of both substitutions on the 10-ethyl-7-oxo-7, 10-dihydropyrido[2,3-*f*]quinoxaline skeleton. Three C-8 unsubstituted quinoxalines **1a**–**c** exhibited emission spectra with broad emission bands, characterized for **1a** with the maxima at 450, 490 and 555 nm (Figure 2b). Despite the comparable molar absorptivities of derivatives **1**–**3a** at the excitation wavelength (350 nm, Figure 2a), a significant decrease in the emission is obvious for the ethyl carboxylate **2a** and almost negligible emission is found for carboxylic acid **3a**, most probably due to the electron-withdrawing properties of the carboxylic acid group [27]. Additionally, the potential interaction of quinoxalines with DMSO solvent possessing a proton accepting capacity (hydrogen bond acceptor basicity of 0.76 [28]) cannot be excluded [29]. Similar behavior was previously detected for 9-ethyl-6,9-dihydro-6-oxo-[1,2,5]selenadiazolo[3,4-*h*]quinoline-7-carboxylic acid [30]. The effect of the C-2 and C-3 di-substitution character on the emission spectra was monitored for the ethyl carboxylate quinoxalines. The derivatives **2a**–**c** reveal emission spectra with two broad absorption maxima in the region 440 and 505 nm, respectively (Figure 2d).

### 2.2. FT-IR Spectroscopy

A detailed analysis of the FT-IR spectra of the quinoxaline derivatives was performed by the procedure of simulation of the corresponding experimental spectra (Table S1). The stretching vibration of the C=O group in the 4-pyridone moiety is observed for all investigated derivatives in the 1632–1620 cm^−1^ range, which is in good accordance with previously published data for 7,10-dihydro-7-oxopyrido[2,3-*f*]quinoxalines [23,24], indolo[3,2-*b*]- and [2,3-*b*]pyrido[2,3-*f*]-quinoxaline-3-carboxylic esters [21], quinolones [31,32] and selenadiazoloquinolones [30]. The presence of substituents at the C-8 position of the 4-pyridone ring (ethyl carboxylate or carboxylic acid) affects the FT-IR spectra more significantly than the substitution at C-2 and C-3 on the pyrazine moiety (methyl or phenyl group). A weak broad band at 3439 cm^−1^ attributed to the O-H stretching vibration was observed for all carboxylic acids **3a**–**3c**, as well as the typical broad band characteristic for carboxylic acid dimers in the region between 2900 and 2600 cm^−1^. The carboxylic acid C=O stretching vibration of **3a**–**3c** occurred in the 1733–1720 cm^−1^ region. The C=O stretching vibration of the ethyl carboxylate group for derivatives **2a**–**2c** differed in the 1726–1698 cm^−1^ region. The shift of this band towards lower wavenumbers can be explained by formation of intermolecular hydrogen bonds of pairwise grouped molecules around the inversion centers in the solid state via the carbonyl group of the ethyl ester and ethyl group at the 4-pyridone moiety as was observed for indolo[3,2-*b*]- and [2,3-*b*]pyrido[2,3-*f*]quinoxaline-3-carboxylic esters [21,22]. The ring carbon-carbon and carbon-nitrogen stretching vibrations occur in the region 1630–1400 cm^−1^, where several overlapping peaks were observed. The phenyl disubstituted quinoxolines **1c**, **2c** and **3c** exhibit two strong absorption bands at ~805 cm^−1^ and ~700 cm^−1^ in accord with vibrations of monosubstituted benzenes (out-of-plane aromatic C–H bending vibration).

### 2.3. Photoinduced Processes of Quinoxaline Derivatives Monitored by EPR Spin Trapping Technique

**Figure 3:**
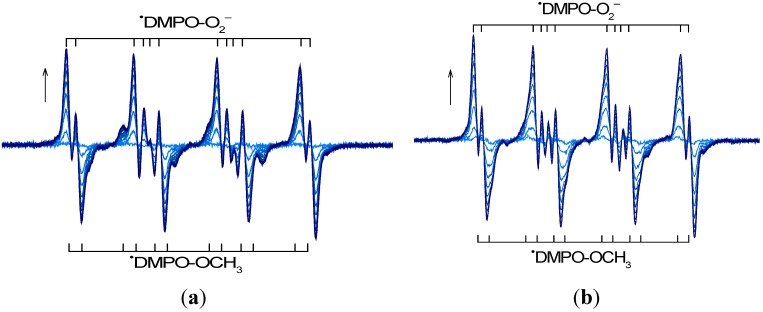
The time evolution of the EPR spectra (sweep width, *SW* = 6 mT) monitored upon UVA photoexcitation (λ_max_ = 365 nm, irradiance 15 mW cm^−2^) of aerated dimethylsulfoxide solutions of (**a**) **1a** and (**b**) **3a** in the presence of the DMPO spin trapping agent. Initial concentrations of quinoxalines *c*_0,Q_ = 0.8 mM; *c*_0,DMPO_ = 0.02 M.

The behavior of the 10-ethyl-7-oxo-7,10-dihydropyrido[2,3-*f*]quinoxaline derivatives upon UVA irradiation was investigated by EPR spectroscopy in order to monitor the generation of reactive paramagnetic intermediates. The photoinduced processes of the heterocyclic compounds are often coupled with the activation of molecular oxygen leading to the generation of reactive oxygen species (ROS) and further reactive radical species, depending on the character of the solvent and atmosphere used [13,30,33]. Consequently, an EPR spin trapping technique was applied to detect and identify the radical species generated upon UVA photoexcitation of the studied derivatives in DMSO or mixed (DMSO/H_2_O, 1:1 v:v) solutions under air or under inert atmosphere. A continuous irradiation (λ_max_ = 365 nm) of the quinoxaline derivatives dissolved in aprotic DMSO under air in the presence of the spin trapping agent 5,5-dimethyl-1-pyrroline *N*-oxide (DMPO) leads to a gradual increase in the EPR signal, as depicted for the **1a** and **3a** derivatives in Figure 3.

Immediately after the irradiation start the twelve-line EPR spectrum arises, assigned to the superoxide radical anion spin adduct ^•^DMPO-O_2_^−^, characterized with well-known spin Hamiltonian parameters summarized in Table 2. As the irradiation continues, another twelve-line signal is developed with the hyperfine coupling constants (hfcc) fully compatible with methoxyl radical added to DMPO. Consequently, the experimental EPR spectrum represents a linear combination of two spin adducts, *i.e.*, ^•^DMPO-O_2_^−^ and ^•^DMPO-OCH_3_ (Figure 3). This indicates that the excited states of the quinoxaline derivative interact with the molecular oxygen via electron transfer processes generating the superoxide radical anion. As was previously observed for various quinolone derivatives, under analogous experimental conditions, the quenching of the photoexcited states of quinolones by the interaction with solvent molecules [17,30], or consecutive reactions of O_2_^•−^ with DMSO lead to the formation of methyl and methoxyl radicals [34].

The results of a detailed simulation analysis of the EPR spectra obtained upon a continuous irradiation in DMSO for selected quinoxalines, depicted in Figure S2 with the spin Hamiltonian parameters of the corresponding spin adducts listed in Table 2, reveal also the presence of further radical species and even the degradation processes on the spin trapping agent DMPO itself.

**Table 2:** Spin Hamiltonian parameters (hyperfine coupling constants and *g*-values) of spin-adducts elucidated from the simulations of experimental EPR spectra obtained upon UVA photoexcitation (λ_max_ = 365 nm) of quinoxaline derivatives in dimethylsulfoxide and dimethylsulfoxide/water (1:1 v:v) in the presence of spin trapping agents.

Spin-Adduct	Hyperfine Coupling Constants (mT)		*g*-Value	Reference
	*a*_N_	*a*_H_		
**DMSO**				
**^•^DMPO-O_2_^−^**	1.283	1.029, 0.136	2.0059	[35,36]
**^•^DMPO-OCH_3_**	1.318	0.825, 0.187	2.0059	[36,37]
**^•^DMPO-OR**	1.426	1.249	2.0057	[37]
**^•^DMPO-CH_3_**	1.472	2.114	2.0056	[36]
**^•^DMPO_degr_**	1.533	-	2.0058	[36]
**^•^ND-CH_3_**	1.422	1.300 (3H)	2.0060	[17,36]
**^•^ND-CD_3_**	1.410	0.193 (3D)	2.0060	[30]
**^•^ND-CR_1_**	1.341	-	2.0061	[38,39]
**^•^ND-(CH_2_)_ar_**	1.368	0.315 (2H)	2.0061	[36,38,39]
**DMSO/H_2_O (1:1 v:v)**				
**^•^DMPO-O_2_^−^/OOH**	1.367	1.091, 0.132	2.0059	[36]
**^•^DMPO-OCH_3_**	1.406	0.969, 0.152	2.0059	[36,37]
**^•^DMPO-OH**	1.449	1.337	2.0059	[40]
**^•^DMPO-CH_3_**	1.550	2.280	2.0056	[36]
**^•^DMPO_degr_**	1.574	-	2.0058	[17,36]
**^•^DMPO-N_3_**	1.427, 0.312	1.355	2.0059	[36,41]
***trans*-^•^EMPO-O_2_^−^/OOH**	1.296	1.068	2.0058	[42]
***trans*-^•^EMPO-OCH_3_**	1.281	0.911	2.0059	[43]
***trans*-^•^EMPO-OH**	1.291	1.179	2.0059	[44]
**^•^EMPO-CH_3_**	1.433	2.088	2.0056	[44]
**^•^EMPO-N_3_**	1.405, 0.224	1.242	2.0057	[45]

Generally, upon UVA photoexcitation of all quinoxaline derivatives in the aerated DMSO solutions, the ^•^DMPO-O_2_^−^ adduct dominates the EPR spectra (Figure 3) and the relative concentration of further spin adducts detected (^•^DMPO-OCH_3_, ^•^DMPO-OR, ^•^DMPO-CH_3_, ^•^DMPO_degr_; hfcc and *g*-values listed in Table 2) is dependent on the structure of the individual quinoxaline and on the irradiation time. Figure S2 shows the experimental EPR spectra obtained upon 15 min exposure of quinoxalines **1a**, **3a**, **1c** and **3c** in DMSO/DMPO/air solutions, along with the simulated spectra calculated as a superposition of the EPR signals of the individual spin adducts considering the depicted relative concentrations.

**Figure 4:**
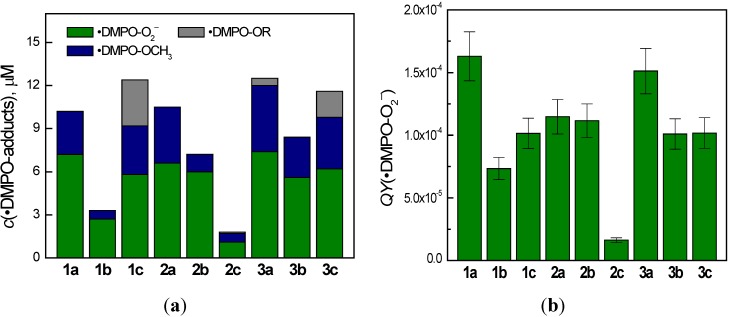
(**a**) Concentrations of the DMPO spin-adducts evaluated by the simulation analysis of the EPR spectra obtained upon 300 s continuous UVA photoexcitation (λ_max_ = 365 nm, total dose 4.5 J cm^−2^) of the quinoxaline derivatives in dimethylsufoxide solutions under air. Initial concentrations of quinoxalines *c*_0,Q_ = 0.8 mM; *c*_0,DMPO_ = 0.02 M; (**b**) Quantum yield of the ^•^DMPO-O_2_^−^ spin-adduct photoinduced generation in the aerated dimethylsulfoxide solutions of quinoxalines in the presence of DMPO spin trapping agent.

Since the superoxide radical anion generated via the photoactivation of molecular oxygen is detected as the corresponding spin adduct ^•^DMPO-O_2_^−^, for the quantitative evaluation of the quinoxaline photoinduced activities, we measured EPR spectra for all the derivatives irradiated with a UVA dose 4.5 J cm^−2^ in DMSO/DMPO/air solutions. The so obtained experimental EPR spectra were analyzed by the simulation procedure and the concentration of individual spin adducts was evaluated (Figure 4a). Subsequently, considering the concentration of ^•^DMPO-O_2_^−^, exposure and the UVA radiation absorbed by the quinoxaline molecules under the given experimental conditions, the quantum yield (*QY*) of the ^•^DMPO-O_2_^−^ generation was calculated in order to indirectly compare the ability of quinoxalines to produce superoxide radical anions (Figure 4b). The highest values of the *QY*(^•^DMPO-O_2_^−^) were found for the derivative **1a** and **3a**, and the lowest value was monitored for the derivative **2c**.

The investigations on the photoinduced processes of quinoxalines in aprotic DMSO solvent were completed by monitoring the radical species formation under an inert atmosphere in the presence of DMPO or nitrosodurene (ND) spin trapping agents. The experimental EPR spectra obtained upon exposure of the derivatives **3b** and **3c**, along with their simulations are illustrated in Figure S3. Upon irradiation of **3b**/DMSO/DMPO/argon solutions we obtained a six-line signal with the spin Hamiltonian parameters well-correlated with ^•^DMPO-CH_3_ spin adduct (Table 2). The application of ND provided unambiguous evidence of methyl radical generation, as the experimental spectrum of the irradiated **3b**/DMSO/ND/argon solution corresponds to the interaction of the unpaired electron with one nitrogen nucleus and three equivalent hydrogen nuclei (hfcc and *g*-value in Table 2). When exchanging DMSO with DMSO-*d_6_* analogue, the EPR spectra fully compatible with ^•^ND-CD_3_ spin adduct were measured (Table 2). This proves that the methyl radicals are generated from the DMSO, most probably via the interactive deactivation of the photoexcited quinolone with the solvent molecules. Besides ^•^ND-CH_3_ spin adduct, the EPR spectrum measured in deoxygenated **3b**/DMSO/ND solution comprised a further spin adduct of lower intensity, with the spin Hamiltonian parameters corresponding to the spin adduct of carbon-centered radical most probably of aromatic origin (Figure S3b, Table 2). The photoexcitation of **3c**/DMSO/DMPO/argon and **3c**/DMSO/ND/argon also results in the production of methyl radical adducts ^•^DMPO-CH_3_ and ^•^ND-CH_3_ (Figure S3c,d). In the presence of ND the spectrum obtained consists of an asymmetrical three-line signal interpreted as a superposition of the carbon-centered spin adduct ^•^ND-CR_1_ and ND^•−^ produced by the electron transfer from the photoexcited quinoxaline molecules to the spin trapping agent (Figure S3d, Table 2).

The photoinduced generation of ROS upon UVA excitation of quinoxalines was performed additionally also in the mixed solvent DMSO/H_2_O (1:1 v:v), since the quinoxaline solubility in aqueous media is limited. To overcome the problem of lower oxygen solubility in DMSO/H_2_O the solutions were saturated with oxygen prior to the measurement. In aprotic solvents the superoxide radical anion is quite stable because the disproportionation reaction to the peroxide dianion O_2_^2−^ is unfavorable [46]. However, in aqueous solutions the reactions with protons is advantageous and hydrogen peroxide is produced Equation (1) [46]:

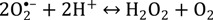
(1)

Upon UVA irradiation hydrogen peroxide is decomposed generating reactive hydroxyl radicals identified as the corresponding spin adducts [47]. The EPR spectra monitored upon the photoexcitation of quinoxalines in DMSO/H_2_O/DMPO/O_2_ solutions are fully compatible with this consideration, since the EPR signals measured correspond to the ^•^DMPO-O_2_^−^/OOH, ^•^DMPO-OH, ^•^DMPO-CH_3_, ^•^DMPO-OCH_3_ spin adducts characterized with hfcc and *g*-values summarized in Table 2. The generation of methyl radicals in mixed solvent may reflect the photoinduced generation of hydroxyl radicals, which react rapidly with DMSO producing ^•^CH_3_[48,49].

**Figure 5:**
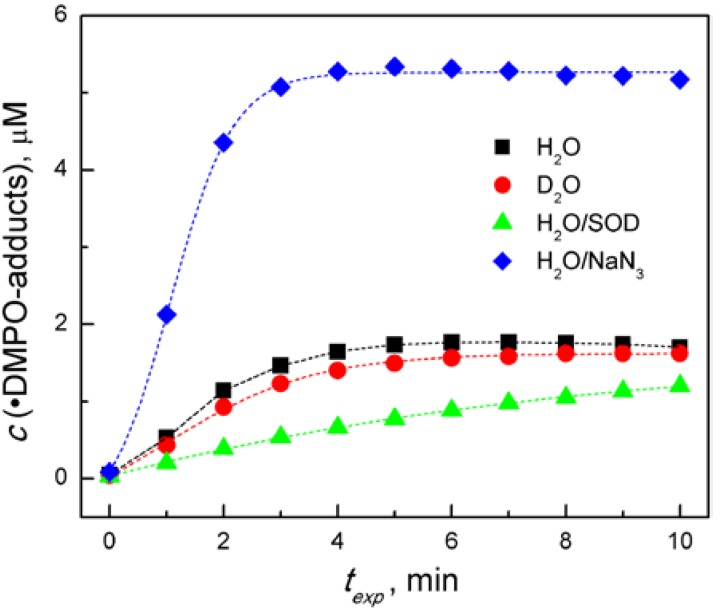
Time dependence of the ^•^DMPO-adducts concentration, generated upon continuous UVA photoexcitation (λ_max_ = 365 nm, irradiance 15 mW cm^−2^) of the aerated solutions of **2b** containing the DMPO spin trapping agent prepared in mixed solvent (DMSO/water, 1:1 v:v), as specified in the legend. Initial concentrations of quinoxalines *c*_0,Q_ = 0.5 mM; *c*_0,DMPO_ = 0.04 M; *c*_0,NaN3_= 0.015 M, *c*_0,SOD_ = 447 units.

In order to confirm the proposed mechanisms we performed a series of EPR experiments with the derivative **2b** in oxygenated mixed solvent containing DMPO and we monitored the effects of an additive on the ^•^DMPO-adducts concentration (Figure 5), as well as on the nature of the individual spin adducts generated (Figure S4). The UVA irradiation of **2b**/DMSO/H_2_O/DMPO/O_2_ results in a gradual increase of the ^•^DMPO-adducts concentration, reaching the steady concentration after 5 min exposure (Figure 5). The simulation of the EPR spectra monitored in this system revealed the superposition of ^•^DMPO-O_2_^−^/OOH, ^•^DMPO-OH, ^•^DMPO-OCH_3_ adducts (Figure S4a). The exchange of water with deuterated water had only a negligible effect on the ^•^DMPO-adduct generation.

However, the addition of a superoxide dismutase (SOD), as a specific enzyme for the superoxide elimination, caused a decrease in the ^•^DMPO-adducts concentration (Figure 5) and the corresponding EPR spectra reveal only two superimposed signals of ^•^DMPO-OH and ^•^DMPO-OCH_3 _adducts (Figure S4b). Sodium azide is well-known as an effective singlet oxygen quencher, however azide anions also react rapidly with hydroxyl radicals producing ^•^N_3_[50]. Indeed, an intense signal of ^•^DMPO-N_3_ spin adducts dominates the EPR spectra measured in the irradiated **2b**/DMSO/H_2_O/DMPO/NaN_3_/O_2_ solutions (Figure 5and Figure S4c). Analogous sets of experiments in the mixed DMSO/H_2_O solvent were performed using the spin trapping agent EMPO, which allows a better stabilization of spin adducts [43]. The results obtained correspond with the above described findings obtained with a standard DMPO spin trapping agent as summarized in Figure S4d–f.

### 2.4. Photoinduced Processes of Quinoxalines in the Presence of Sterically Hindered Amines

**Figure 6:**
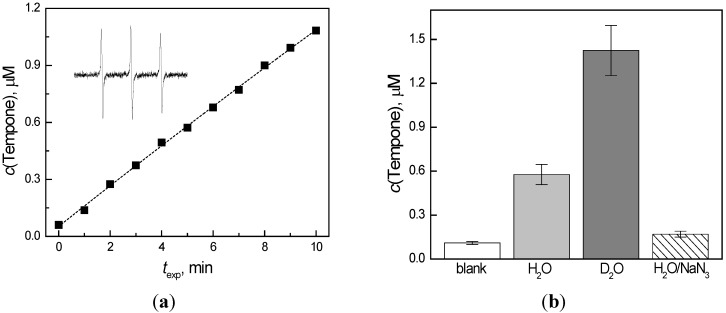
(**a**) The time dependence of Tempone concentration measured upon continuous photoexcitation (λ_max_ = 365 nm; irradiance 15 mW cm^−2^) of oxygen-saturated DMSO/H_2_O (1:1 v:v) solutions of derivative **2b** in the presence of sterically hindered amine TMPO. Inset: The experimental EPR spectrum (*SW* = 6 mT) monitored upon photoexcitation of oxygen-saturated **2b**/DMSO/H_2_O/TMPO. Initial concentrations of **2b** *c*_0,**2b**_ = 0.5 mM; *c*_0,TMPO_ = 0.01 M; *c*_0,NaN3_= 0.015 M; (**b**) Tempone concentration monitored upon 300 s irradiation (total dose 4.5 J cm^−2^) in oxygen-saturated solution of **2b** containing TMPO in dimethylsulfoxide mixed solvent (DMSO/water, 1:1 v:v) as specified in the legend.

Generation of the superoxide radical anion via electron transfer from the excited state of the quinoxaline molecule to the molecular oxygen was confirmed by the spin trapping technique and described above. However the photoinduced activation of molecular oxygen is usually coupled with both electron and energy transfer mechanisms, by which O_2_^•−^ and singlet oxygen are generated, respectively. The formation of ^1^O_2_ was monitored upon continuous, *in situ*, irradiation (λ_max_ = 365 nm) of the quinoxaline derivatives in the presence of the sterically hindered amine 4-oxo-2,2,6,6-tetramethylpiperidine (TMPO). The method, based on the oxidation of the amine hydrogen at the tetramethylpiperidine moiety of the TMPO molecule by the singlet oxygen, generating a semi-stable nitroxide radical 4-oxo-2,2,6,6-tetramethylpiperidine *N*-oxyl (Tempone), has been widely applied in the investigations of molecular oxygen activation in the presence of various nitrogen heterocyclic compounds [17,30,31,51,52]. According to our recent investigations on the oxidation of sterically hindered amines under various experimental conditions, the careful choice of experimental conditions is crucial for the successful application of this method; mainly considering the selectivity [41]. In the experiments in DMSO, the irradiation of the quinoxaline/DMSO/TMPO/air mixtures resulted in an immediate increase of a three-line signal, which reached its maximum within a few minutes and quickly decreased (data not shown). Similar behavior was observed previously in studies on various quinolone derivatives [31] and can be explained by the simultaneous generation of superoxide radical anions, stabilized in aprotic DMSO, and further oxygen-, carbon-centered paramagnetic species, which interact with Tempone, generating diamagnetic products, reflected by a decrease of the Tempone EPR signal [41]. Due to the problems with the solubility of the studied derivatives in water, mixed DMSO/H_2_O solvent was applied in further experiments. However the presence of water in the system leads to the generation of hydroxyl radicals, which can also participate in the oxidation of TMPO to Tempone. Upon the continuous irradiation of the system quinoxaline/DMSO/H_2_O/TMPO/O_2_a gradual increase of a three-line signal, characteristic for the Tempone nitroxide radical (*a*_N_ = 1.56 mT, *a*_13C_(4^13^C) = 0.58 mT, *a*_13C_(2^13^C) = 0.25 mT; *g* = 2.0057) was observed, as depicted for the **2b** derivative in Figure 6a. The concentration of Tempone generated after a 300 s exposure (λ_max_ = 365 nm) in the systems of a different composition is shown in Figure 6b.

The presence of the quinoxaline in the TMPO solution obviously showed the formation of Tempone radical, as in the irradiated solutions without quinoxaline only negligible oxidation of TMPO was found (blank in Figure 6b). The exchange of water with its deuterated analogue resulted in a significant increase in the Tempone concentration (Figure 6b). The addition of a deuterated solvent can improve the yield of the photogenerated nitroxide radicals and thus provide the evidence for ^1^O_2_ involvement in SHA oxidation. The lifetime of singlet oxygen in D_2_O (~60 μs) is significantly higher than in H_2_O (~4.0 μs) [53,54], consequently if the reaction in aqueous solutions is dependent on ^1^O_2_, application D_2_O instead of H_2_O should potentiate the reaction. Theoretically, a type I photooxidation process would be unaffected [50]. The experiments performed in DMSO/D_2_O (1:1 v:v) solutions with DMPO spin trap, monitoring electron transfer process to molecular oxygen (type I photooxidation) confirmed, that the concentration of ^•^DMPO-adducts was identical as in DMSO/H_2_O (1:1 v:v.) (Figure 5). On the other hand, the oxidation of TMPO to Tempone was substantially improved in the mixed solvent containing deuterated water (Figure 6b), evidencing indirectly the participation of singlet oxygen in the TMPO oxidation. The amount of Tempone generated was diminished almost to the value obtained in the blank experiment, *i.e.*, without the quinoxaline derivative, as the sodium azide was added to the system. Even though the NaN_3_ is a well-known singlet oxygen quencher [55], its reaction with the hydroxyl radicals cannot be excluded as was shown in spin trapping experiments (Figure 5 and Figure S4c,f).

**Figure 7:**
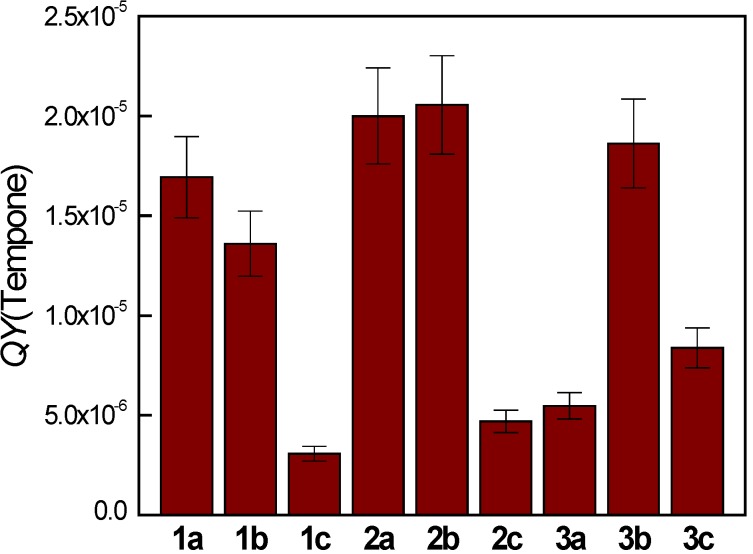
Quantum yield of photoinduced Tempone generation evaluated for the photoexcitation (λ_max_ = 365 nm; irradiance 15 mW cm^−2^, total dose 4.5 J cm^−2^) of quinoxalines in DMSO/H_2_O (1:1 v:v) solutions saturated with oxygen.

To quantify the ability of the quinoxaline derivatives investigated to generate Tempone upon irradiation (λ_max_ = 365 nm) in oxygen-saturated DMSO/H_2_O solutions in the presence of TMPO, quantum yield was evaluated as shown in Figure 7.

### 2.5. Quinoxaline Photoexcitation Monitored by UV/Vis Spectroscopy — Steady State Experiments

**Figure 8:**
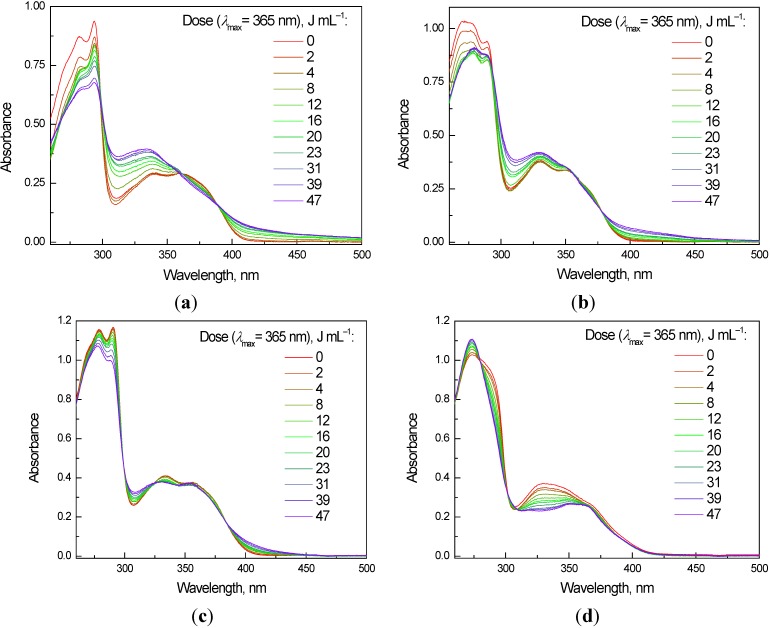
Changes in the electronic absorption spectra monitored upon steady-state monochromatic irradiation (λ_max_ = 365 nm, irradiance 13 mW cm^−2^) of the aerated 0.05 mM solutions of (**a**,**c**) **2a** and (**b**,**d**) **3a** in (**a**,**b**) DMSO or in (**c**,**d**) mixed solvent DMSO/H_2_O (1:1 v:v); optical path length 1 cm.

Generation of reactive radical intermediates upon the irradiation of the studied compounds in aprotic solutions was confirmed. Along with the reactive oxygen species identified via the EPR spin trapping technique the radical intermediates originating from the interactions of the molecules’ excited states with the solvent were also observed, suggesting a more complex reaction mechanism initiated by the photoexcited quinoxaline molecules. Therefore we performed a series of UV/vis steady state experiments, where the photochemical stability of the quinoxaline derivatives was monitored by recording the electronic absorption spectra of their solutions in the aprotic (DMSO) or mixed solvent (DMSO/water, 1:1 v:v) after a defined exposure. Previous studies on different quinolone derivatives have shown only negligible changes in the UV/vis spectra upon UVA exposure under the applied experimental conditions, reflecting a high photochemical stability [30]. The quinoxaline derivatives **1a**–**c**, without the substitution at the C-8, exhibited only minor changes in the electronic spectra upon the discontinuous irradiation (λ_max_ = 365 nm), even after 60 min exposure (dose of 47 J mL^−1^) in both DMSO and mixed solvent (data not shown). Almost no changes in the absorption spectra upon irradiation were observed for the 2,3-diphenyl substituted derivatives **1c**, **2c** and **3c**. This suggests a rather high photochemical stability of these derivatives indicating that no significant damage to the quinoxaline molecules occurs under the given experimental conditions, similar to those applied by the EPR photochemical experiments. The analogous experiments with the C-8 substituted derivatives **2a**,**b** and **3a**,**b** brought different results. Here, the UVA exposure initiated changes in the UV/vis spectra, namely a decrease of the absorption bands intensity in the region 275–300 and at 375 nm along with an increase of the bands at 330 nm and 400 nm. The changes in the spectra proportional to the irradiation dose are more evident in the aprotic DMSO solvent, as shown in Figure 8 for the **2a** and **3a** derivatives. The formation of new absorption bands at higher wavelengths and simultaneous decrease in other absorption bands (e.g., isosbestic points at 388, 360 and 299 nm found for **2a** in DMSO, Figure 8a) can be explained by the generation of new intermediates via the interaction of the quinoxaline molecule with the reactive oxygen species generated upon UVA irradiation. However, we cannot exclude such reactions also for the derivatives **1a**–**c**, due to the negligible changes in the UV/vis spectra of oxidized quinoxalines [1].

## 3. Experimental Section

The investigated derivatives of 10-ethyl-7-oxo-7,10-dihydropyrido[2,3-*f*]quinoxaline (Figure 1) were prepared as described in the literature [56]. The stock solutions of quinoxaline were freshly prepared for each series of experiments in dried DMSO (Merck, Darmstadt, Germany, SeccoSolv^®^, max. 0.05% H_2_O) or in mixed solvent (DMSO/water 1:1 v:v) at a concentration of 1 mM. To increase the solubility of derivative **3c** both in DMSO and in the mixed solvent, the addition of an equimolar amount of NaOH was necessary. The spin trapping agent 5,5-dimethyl-1-pyrroline *N*-oxide (DMPO; Sigma-Aldrich, Buchs, Switzerland) was distilled prior to the application. Other spin traps, *i.e.*, 2,3,5,6-tetramethylnitrosobenzene (nitrosodurene, ND; Aldrich) and 5-(ethoxycarbonyl)-5-methyl-1-pyrroline *N*-oxide (EMPO; Enzo Life Sciences, Farmingdale, NY, USA), were used without extra purification. All spin trapping agents were stored at −18 °C. The stock solutions of the spin traps were prepared in DMSO, apart from the ND, characteristic with a limited solubility in DMSO, which was applied in a saturated solution directly before the specific experiment. SOD (Sigma-Aldrich, from bovine erythrocyte, specific activity 4470 units/mg) was used for the target termination of superoxide radical anions, and sodium azide (analytical grade, Sigma-Aldrich) for singlet oxygen quenching.

The formation of paramagnetic intermediates upon UVA irradiation of the 10-ethyl-7-oxo-7, 10-dihydropyrido[2,3-*f*]quinoxaline derivatives studied was monitored *in situ* by a well-established EPR spin trapping technique [57]. The photoinduced production of singlet oxygen during the excitation of quinoxalines was followed by the oxidation of a stericaly hindered amine 4-oxo-2,2,6,6-tetramethylpiperidine (TMPO, Merck-Schuchardt, Hohenbrunn, Germany) [51,52]. The solution of a quinoxaline derivative containing a spin trapping agent or the TMPO was mixed and carefully saturated with air, argon or oxygen using a slight gas stream immediately before the EPR measurement. The so prepared sample was transferred to a small quartz flat cell (WG 808-Q, optical cell length 0.04 cm; Wilmad-LabGlass, Vineland, NJ, USA) optimized for the TE_102_ cavity (Bruker, Rheinstetten, Germany) of the X-band EPR spectrometer (EMX Plus, Bruker). During the EPR photochemical experiments the samples were irradiated at 295 K directly in the EPR resonator, and the EPR spectra were recorded *in situ* during continuous photoexcitation or after a defined exposure. As an irradiation source a UV LED monochromatic radiator (λ_max_ = 365 nm; bluepoint LED, Hönle UV Technology, Gräfelfing/München, Germany) was used. The irradiance value (λ_max_ = 365 nm; 15 mW cm^−2^) within the EPR cavity was determined using a UVX radiometer (UVP, 360 USA). The *g*-values were determined using a built-in magnetometer. The EPR spectra obtained were analyzed and simulated using the Bruker software WinEPR and SimFonia and the Winsim 2002 software [58]. The concentrations of the detected paramagnetic species were determined using the solutions of 4-hydroxy-2,2,6,6-tetramethylpiperidine *N*-oxyl and 4-oxo-2,2,6,6-tetramethylpiperidine *N*-oxyl (Aldrich) as calibration standards.

The UV/visible spectra of the quinoxaline derivatives dissolved in DMSO or in mixed solvent DMSO/water (1:1 v:v) were recorded by a UV-3600 UV-vis-NIR spectrophotometer (Shimadzu, Kyoto, Japan) with a 1-cm square quartz cell. Changes in the electronic spectra upon discontinuous irradiation were monitored for freshly prepared quinoxaline solutions irradiated under air in a 1 cm quartz cell using a monochromatic LED source at 365 nm. The first spectrum was measured without irradiation, and then the spectra were recorded immediately after a defined exposure, until the overall exposure time reached 60 min, which corresponds to a 47 J mL^−1^ radiation dose.

Fluorescence emission spectra of the 0.25 mM quinoxaline solutions in aerated DMSO were recorded using a Fluorolog 3 (Horiba Jobin Yvon, Edison, NJ, USA) spectrofluorimeter or a Perkin-Elmer LS 50 luminescence spectrometer (Waltham, MA, USA). The excitation wavelength, 350 nm, was chosen corresponding to the λ_max_ absorption value from the UV/vis spectroscopy investigations. The emission spectra were recorded in a 1-cm quartz spectrophotometer cell using a perpendicular arrangement from 380 to 670 nm.

Infrared spectra of the quinoxaline derivatives in the region 4000–400 cm^−1^ were recorded with a Nicolet model NEXUS 470 FT-IR spectrometer (Thermo Fisher Scientific Inc., Waltham, MA, USA) at room temperature using KBr pellets. The crystalline sample was thoroughly mixed with KBr (for IR spectroscopy, Fluka, Buchs, Switzerland) and so prepared mixture was pressed into a pellet. Simulations of the selected spectra were performed by Thermo Scientific Peak Resolve (included in Omnic 7.4 Thermo Fisher Scientific Inc., Waltham, MA, USA), which fits a number of individual synthetic peaks to a complex set of overlapping peaks in a spectrum.

All quantum chemical calculations were performed using the Gaussian 03 program package [59]. The optimal geometries of quinoxaline derivatives were obtained using the density functional theory (DFT) method with B3LYP (Becke’s three parameter Lee-Yang-Parr) functional without any constraints. The solvent effect of DMSO was included using the IEF-PCM (Polarizable Continuum Model based on the integral equation formalism variant) approach [60]. Based on the optimized geometries, the vertical electronic transition energies and the oscillator strengths between the initial and final excited states were computed by the time-dependent (TD)-B3LYP method [61].

## 4. Conclusions

**Scheme 1:**
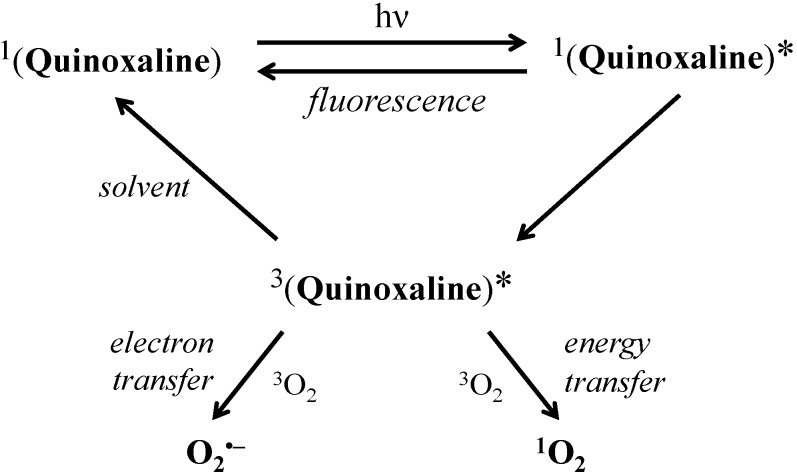
The proposed photoinduced processes occurring upon the irradiation (λ_max_ = 365 nm) of the studied quinoxaline derivatives in aprotic solvent.

EPR spin trapping experiments evidenced the dominant generation of superoxide radical anions by the photoexcitation of the studied quinoxaline derivatives in the aprotic DMSO. Other oxygen- and carbon-centered radical intermediates detected as the corresponding spin adducts originate mainly from DMSO, in accord with previous photoinduced studies in this solvent. In mixed DMSO/H_2_O media besides the superoxide hydroxyl radicals are also produced. Simultaneous generation of singlet oxygen upon the photoexcitation of the quinoxaline derivatives in the mixed solvent was proved by the oxidation of the sterically hindered amine TMPO to Tempone. The results confirm the activation of molecular oxygen by the excited states of the quinoxaline derivatives via both electron and energy transfer mechanisms (Scheme 1) and thus indicate the photosensitizer character of the studied derivatives. Photochemical activity of the individual derivatives upon UVA irradiation (λ_max_ = 365 nm) was quantified by evaluating the quantum yield of the ^•^DMPO-O_2_ and Tempone generation.

## Supplementary Material

Supplementary materials can be accessed at: http://www.mdpi.com/1420-3049/19/8/12078/s1.

## Supplementary Files

Supplementary File 1
