# Supplementary material for: Fused-Ring Derivatives of Quinoxalines: Spectroscopic Characterization and Photoinduced Processes Investigated by EPR Spin Trapping Technique
Source: Molecules. 2014 Aug 12;19(8):12078–98. doi: 10.3390/molecules190812078 (PMC6271815; doi:10.3390/molecules190812078)

Supplementary Material

Table S1. Selected vibration frequencies (in cm^{-1}) of investigated quinoxalines **1a**–**3c** obtained from FT-IR spectra.

Compd.	$\tilde{\nu}$ (cm^{-1})
**1a**	3047, 2995, 2966, 1626 (C=O), 1588, 1500, 1466, 1401, 1351, 1252, 1180, 1131, 1116, 1088, 1075, 1036, 953, 850, 837, 827, 797, 741, 706, 471
**1b**	3048, 2983, 2939, 1620 (C=O), 1574, 1496, 1464, 1405, 1378, 1266, 1237, 1197, 1166, 1138, 1115, 833, 799, 741, 731, 483
**1c**	3058, 2989, 2927, 1632 (C=O), 1585, 1490, 1460, 1444, 1357, 1248, 1201, 1175, 1157, 1091, 1075, 1023, 825, 806, 776, 742, 701, 588, 467
**2a**	3056, 2986, 2927, 1724 (C=O, ester), 1623 (C=O), 1590, 1498, 1462, 1411, 1386, 1358, 1309, 1241, 1173, 1121, 1083, 1032, 825, 784, 743, 477
**2b**	3037, 2981, 2931, 1726, 1695 (C=O, ester), 1623 (C=O), 1592, 1490, 1457, 1376, 1365, 1320, 1294, 1230, 1199, 1175, 1118, 1077, 1030, 846, 822, 787, 714, 678, 481
**2c**	3058, 2984, 2929, 1726, 1685 (C=O, ester), 1636 (C=O), 1612, 1590, 1534, 1480, 1452, 1365, 1355, 1318, 1245, 1224, 1194, 1126, 1096, 1077, 1018, 802, 782, 765, 703, 697, 474
**3a**	3435 (OH), 3059, 2983, 2603 (dimer), 1720 (C=O, acid), 1619 (C=O), 1541, 1522, 1465, 1439, 1374, 1352, 1260, 1228, 1188, 1122, 1042, 872, 827, 793, 768, 479
**3b**	3439 (OH), 3052, 2991, 2614 (dimer), 1733 (C=O, acid), 1621 (C=O), 1556, 1536, 1443, 1375, 1335, 1256, 1234, 1188, 1120, 1091, 1006, 882, 846, 824, 789, 719, 482
**3c**	3439 (OH), 3039, 2610 (dimer), 1724 (C=O, acid), 1620 (C=O), 1597, 1533, 1513, 1473, 1458, 1445, 1376, 1357, 1315, 1277, 1255, 1227, 1202, 1095, 1027, 805, 783, 768, 699, 606, 480

Figure S1. Plots of the B3LYP(IEFPCM = DMSO) molecular orbitals contributing to the selected optical transitions of quinoxaline derivatives (λ represents the calculated absorption maximum and *f* its oscillator strength): (**a**) **1a**; (**b**) **1c**. The depicted isosurface value is 0.035 a.u.

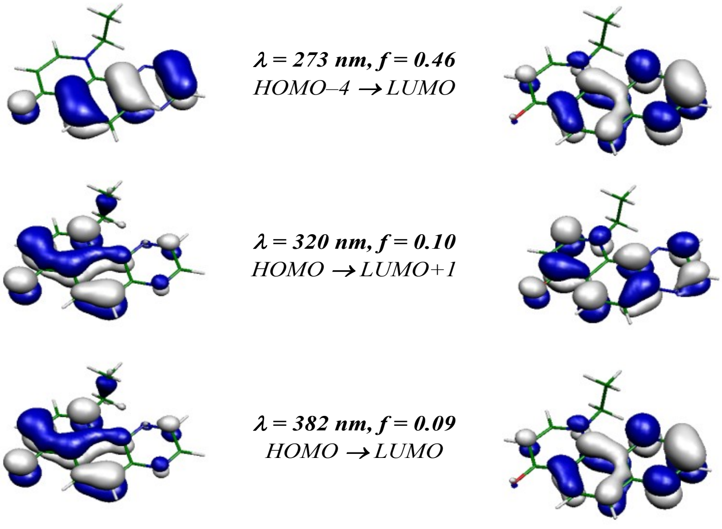

(**a**)

Figure S1. *Cont.*

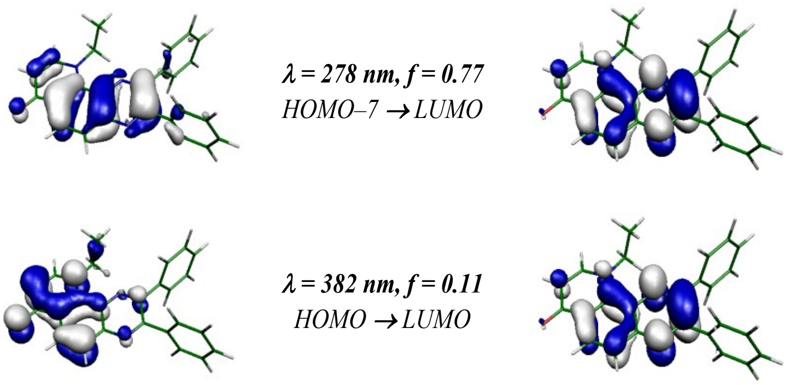

(**b**)

Figure S2. Experimental (black) and simulated (red) EPR spectra (*SW* = 7 mT) obtained upon irradiation (λ_{max} = 365 nm; irradiance 15 mW cm^{-2}) of the aerated dimethylsulfoxide solutions of (**a**) **1a**, (**b**) **3a**, (**c**) **1c** and (**d**) **3c** in the presence of DMPO spin trapping agent. Initial concentrations of quinoxalines $c_{0,Q}$ = 0.8 mM; $c_{0,DMPO}$ = 0.02 M. [Solution of **3c** contains equimolar amount of NaOH in DMSO/water (200:1 v:v)]. Simulations represent linear combinations of the corresponding spin-adducts (hfcc parameters listed in Table 2): (**a**) $^{\bullet}$DMPO-O_2^- (relative concentration in %; 74), $^{\bullet}$DMPO-OCH_3 (22) and $^{\bullet}$DMPO-OR (4); (**b**) $^{\bullet}$DMPO-O_2^- (51), $^{\bullet}$DMPO-OCH_3 (41), $^{\bullet}$DMPO-OR (6), $^{\bullet}$DMPO-CH_3 (1.5) and $^{\bullet}DMPO_{degr}$ (0.5); (**c**) $^{\bullet}$DMPO-O_2^- (78), $^{\bullet}$DMPO-OCH_3 (8), $^{\bullet}$DMPO-OR (7), $^{\bullet}$DMPO-CH_3 (6) and $^{\bullet}DMPO_{degr}$ (1); (**d**) $^{\bullet}$DMPO-O_2^- (62), $^{\bullet}$DMPO-OCH_3 (27) and $^{\bullet}$DMPO-OR (11).

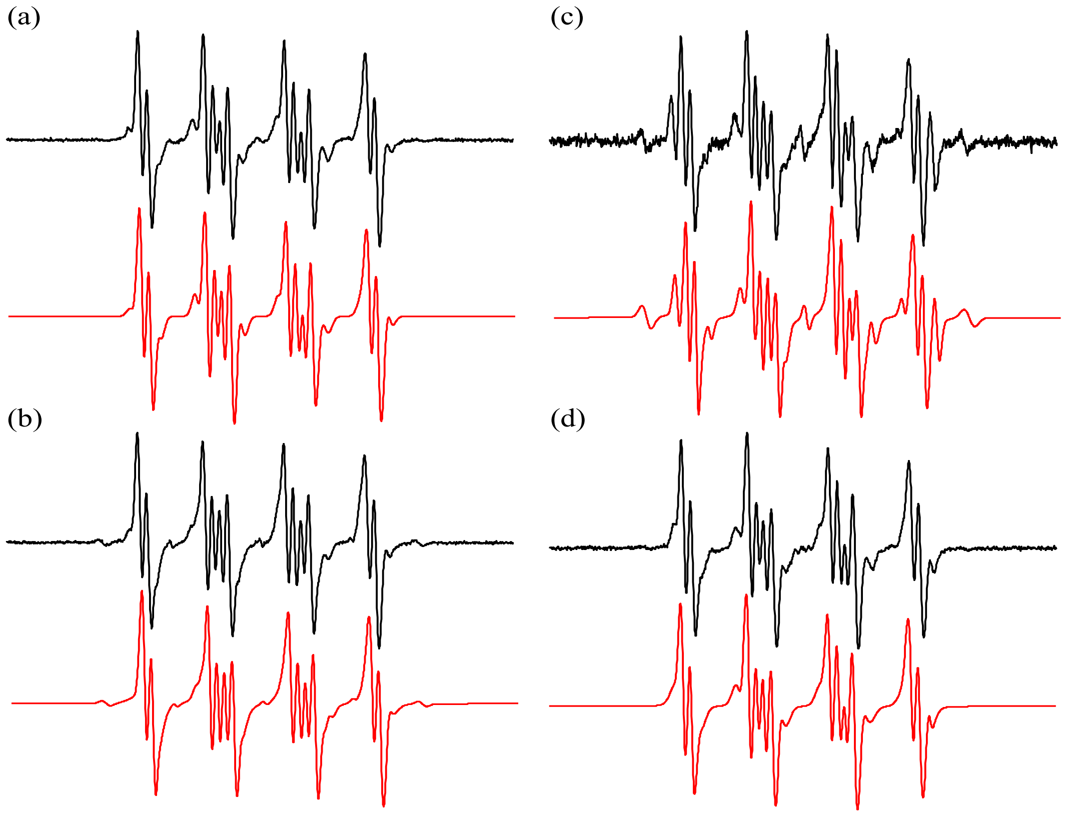

Figure S3. Experimental (black) and simulated (red) EPR spectra (*SW* = 8 mT) obtained upon irradiation (λ_{max} = 365 nm; irradiance 15 mW cm^{-2}) of the argon saturated DMSO solutions of (**a**,**b**) **3b** and (**c**,**d**) **3c** in the presence of (**a**,**c**) DMPO or (**b**,**d**) ND spin trapping agent. Initial concentrations of quinoxalines $c_{0,Q}$ = 0.8 mM; $c_{0,DMPO}$ = 0.02 M; $c_{0,ND}$ ~10 mg mL^{-1}. [Solution of **3c** contains equimolar amount of NaOH in DMSO/water (200:1 v:v)]. Simulations represent linear combinations of the corresponding spin-adducts (hfcc parameters listed in Table 2): (**a**) $^{\bullet}$DMPO-CH_3 (relative concentration in %; 100); (**b**) $^{\bullet}$ND-CH_3 (88) and $^{\bullet}$ND-$(CH_2)_{ar}$ (12); (**c**) $^{\bullet}$DMPO-CH_3 (100); (**d**) $^{\bullet}$ND-CH_3 (83); $^{\bullet}$ND-CR_1 (15) and $ND^{\bullet-}$ (2; a_N = 1.378 mT; g = 2.0061).

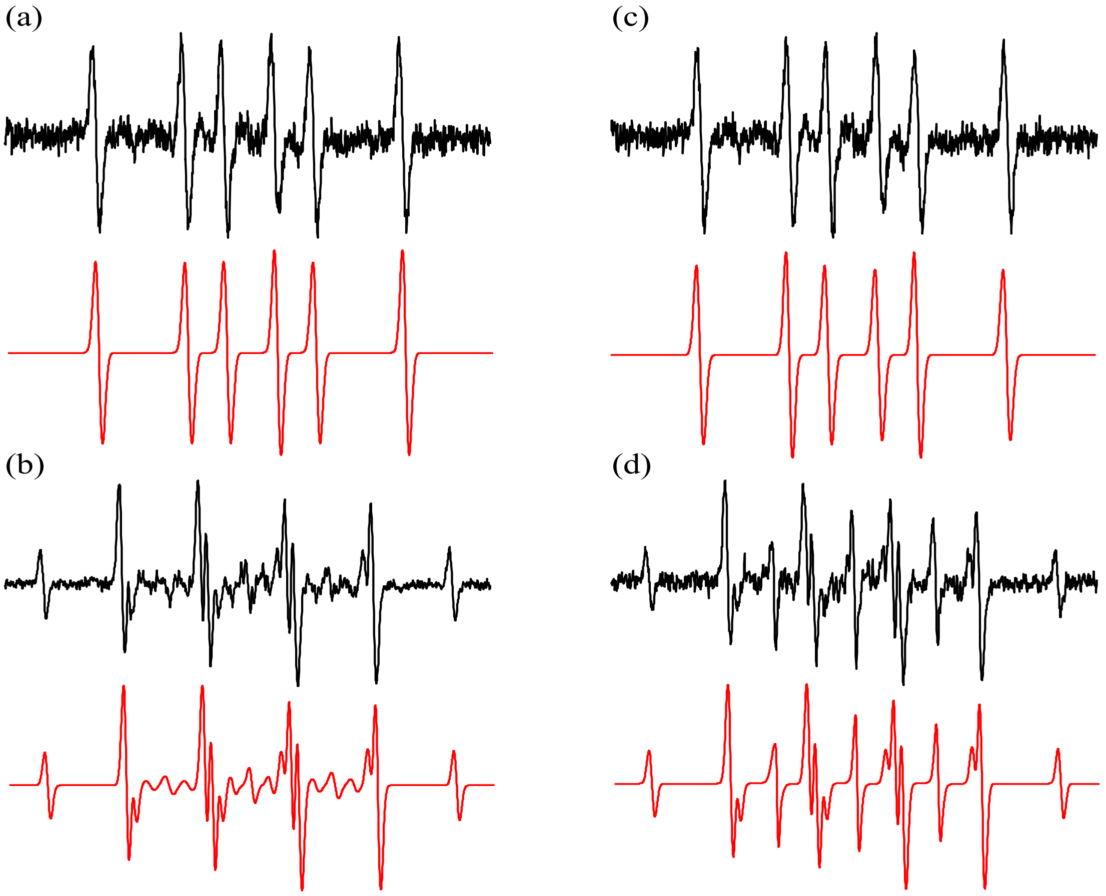

Figure S4. Experimental (black) and simulated (red) EPR spectra (*SW* = 7 mT) obtained after 15 min of irradiation (λ_{max} = 365 nm; irradiance 15 mW cm^{-2}) of the aerated solutions of **2b** in mixed solvent DMSO/H_2O (1:1 v:v) containing DMPO or EMPO spin trapping agent with the addition of SOD or sodium azide. (**a**) **2b**/DMPO; (**b**) **2b**/DMPO/SOD; (**c**) **2b**/DMPO/NaN_3; (**d**) **2b**/EMPO, (**e**) **2b**/EMPO/SOD; (**f**) **2b**/EMPO/NaN_3. Initial concentrations of quinoxalines $c_{0,Q}$ = 0.5 mM; $c_{0,DMPO}$ = 0.04 M; $c_{0,NaN3}$ = 0.015 M, $c_{0,SOD}$ = 447 units. Simulations represent linear combinations of the corresponding spin-adducts (hfcc parameters listed in Table 2): (**a**) $^{\bullet}$DMPO-O_2^-/OOH (relative concentration in %; 58), $^{\bullet}$DMPO-OCH_3 (18) and $^{\bullet}$DMPO-OH (19); $^{\bullet}$DMPO-CH_3 (3) and $^{\bullet}$$DMPO_{degr}$ (2); (**b**) $^{\bullet}$DMPO-OH (56) and $^{\bullet}$DMPO-OCH_3 (44); (**c**) $^{\bullet}$DMPO-OH (34); $^{\bullet}$DMPO-OCH_3 (14) and $^{\bullet}$DMPO-N_3 (52); (**d**) *trans*-$^{\bullet}$EMPO-O_2^-/OOH (44), *trans*-$^{\bullet}$EMPO-OCH_3 (13), *trans*-$^{\bullet}$EMPO-OH (38) and $^{\bullet}$EMPO-CH_3 (5); (**e**) *trans*-$^{\bullet}$EMPO-O_2^-/OOH (16), *trans*-$^{\bullet}$EMPO-OCH_3 (14), *trans*-$^{\bullet}$EMPO-OH (21) and $^{\bullet}$EMPO-CH_3 (49). (**f**) *trans*-$^{\bullet}$EMPO-OH (49), *trans*-$^{\bullet}$EMPO-OCH_3 (23), $^{\bullet}$EMPO-N_3 (22) and $^{\bullet}$EMPO-CH_3 (6).

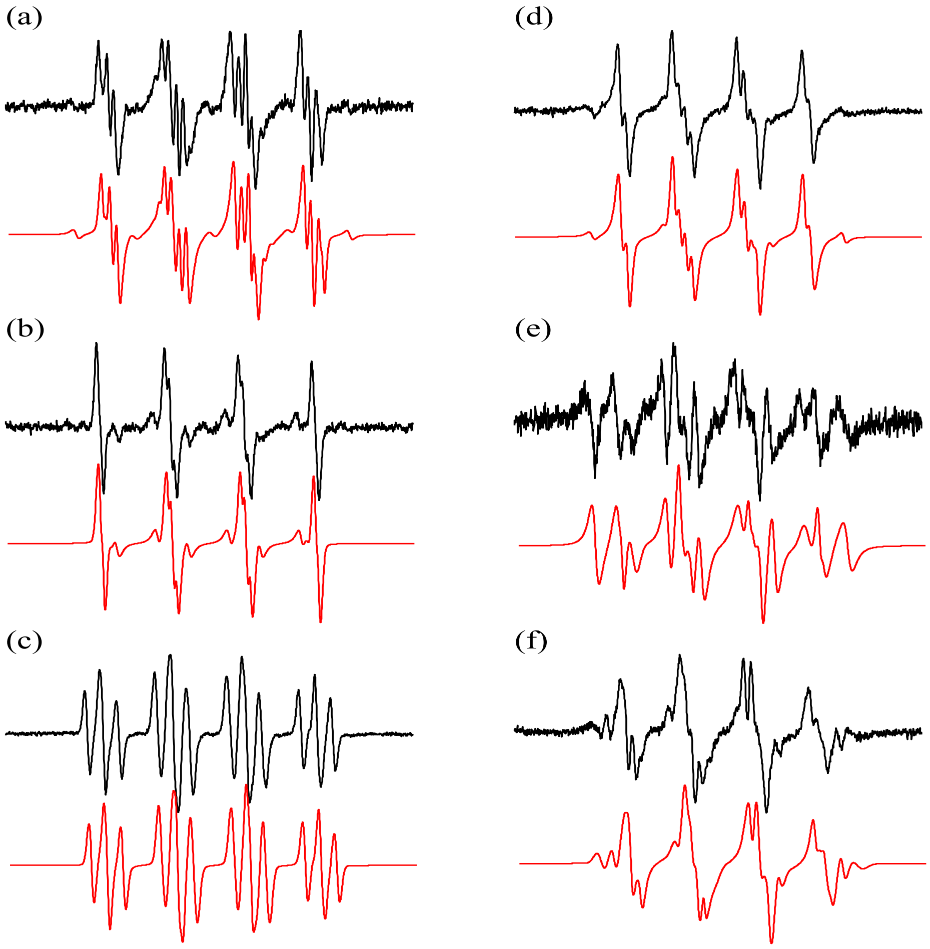

Supplement: Supplementary File 1 [file molecules-19-12078-s001.pdf]
